# Investigation of AC-Measurements of Epoxy/Ferrite Composites

**DOI:** 10.3390/nano10030492

**Published:** 2020-03-09

**Authors:** Moustafa A. Darwish, Alex V. Trukhanov, Oleg S. Senatov, Alexander T. Morchenko, Samia A. Saafan, Ksenia A. Astapovich, Sergei V. Trukhanov, Ekaterina L. Trukhanova, Andrey A. Pilyushkin, Antonio Sergio B. Sombra, Di Zhou, Rajshree B. Jotania, Charanjeet Singh

**Affiliations:** 1 Department of Technology of Electronics Materials, National University of Science and Technology “MISiS”, Leninskii av., Moscow 4119049, Russia; mostafa_ph@science.tanta.edu.eg (M.A.D.); truhanov86@mal.ru (A.V.T.); senatov.os@gmail.com (O.S.S.); dratm@mail.ru (A.T.M.); el_trukhanova@mail.ru (E.L.T.); apilyushkin@gmail.com (A.A.P.); 2 Physics Department, Faculty of Science, Tanta University, Al-Geish st., Tanta 31527, Egypt; samiasaafan@science.tanta.edu.eg; 3 Scientific and Educational Center “Nanotechnology”, South Ural State University, Lenin av. 76, Chelyabinsk 454080, Russia; 4 SSPA “Scientific and Practical Materials Research Centre of the NAS of Belarus”, P. Brovki Str. 19, 220072 Minsk, Belarus; ks-sd@mail.ru; 5 Department of Physics, Federal University of Ceara, Fortaleza, Ceara 60-455-970, Brazil; asbsombra@gmail.com; 6 Electronic Materials Research Laboratory, Key Laboratory of the Ministry of Education & International Center for Dielectric Research, School of Electronic Science and Engineering, Xi’an Jiaotong University, Xi’an 710049, China; zhoudi1220@gmail.com; 7 Department of Physics, Electronics and Space Science, Gujarat University, Gujarat, Ahmedabad 380009, India; rbjotania@gmail.com; 8 School of Electronics and Electrical Engineering, Lovely Professional University, Phagwara, Punjab 144411, India; rcharanjeet@gmail.com

**Keywords:** ferrites, epoxy, nanoparticles, composites, ac-conductivity, dielectric constant, dielectric loss

## Abstract

A pure ferrite and epoxy samples as well as the epoxy/ferrite composites with different 20 wt.%, 30 wt.%, 40 wt.%, and 50 wt.% weight ferrite contents have been prepared by the chemical co-precipitation method. AC-conductivity and dielectric properties such as the dielectric constant and dielectric loss of the prepared samples have been studied. The obtained results showed that the samples had a semiconductor behavior. The dielectric constant of the composites has been calculated theoretically using several models. For the composite sample that contains 20 wt.% of ferrites, these models give satisfactory compliance, while for the composite samples with a higher percentage of nanofillers, more than 30 wt.% theoretical results do not coincide with experimental data. The investigated polymer has very low conductivity, so this type of polymer can be useful for high-frequency applications, which can reduce the losses caused by eddy current. Thus, the prepared samples are promising materials for practical use as elements of microwave devices.

## 1. Introduction

In recent years, nanotechnology has continued to be an essential topic for many researchers around the world due to new phenomena and distinctive properties shown by nanoparticles [1]. Nanotechnology can be an excellent tool in the study, preparation, research, and use of new perspective materials, as well as in solving the problem of particles sizes in the range of 1–100 nm, where unusual phenomena allow new applications [2,3,4]. Tiny nanoparticles with diameters of some nanometers usually look like molecules. Therefore, the atomic and electronic structures of such small nanoparticles possess different features, certainly not the same as those of the bulk materials. The size-dependent abilities of nanoparticles include electronic, magnetic, optical, and chemical substance characteristics. Nanoparticles can be crystalline or amorphous [5].

Nanosized ferrites could be prepared by many methods, including mechanized milling, ultrasonic cavitation method, radio rate of recurrence plasma, hydrothermal technique, reverse micelle method, sol-gel technique, citrate precursor, and co-precipitation technique [6,7,8,9,10,11,12,13,14,15,16,17,18,19,20,21]. It is advisable to possess a synthesis method that allows for governor on the nanoparticle dimension and produces nanoparticles having a significant size distribution [2]. The chemical substance co-precipitation method is the most excellent ideal for the formation of nanoparticles due to its ease and much-improved handle over the crystallite dimension and other qualities of the components [22]. Also, lately, magnetic composites comprising magnetic materials alongside polymers comprise a new era of multifunctional components that mix the properties from the constituents; these components are known as magneto-polymeric components [23].

The characteristics of ferrites are highly susceptible to methodical handling, sintering conditions, and impurity. Nickel-zinc ferrite is one of the first widely used ferrite produced for an open range of applications that recognizes to high saturation magnetization, high resistance, high Curie temperature, high chemical stability, low coercive power, and biodegradability [24,25,26,27,28]. In technological applications as telecommunications, transformers, and microwave devices, etc., Ni-Zn ferrites now play a crucial role. Spinel Ni-Zn ferrites are commonly employed in several devices as core materials, high-frequency transformer-core ferrites, frequency antennas, RAMs (radar absorption materials), and electrical components [29,30,31,32]. Lithium ferrite substituted with zinc has extended importance because it possesses high saturation magnetization, which is found to be suitable in memory core applications and microwave communication [29].

Many researchers work on the 2-phase dielectric-polymer, or magnetic-polymer composites are extensively studied within the last years. Nonetheless, the principal focus is on enhancing the dielectric and magnetic properties. Within the recent past, however, the developments of the three-phase dielectric, dielectric and magnetic polymer polymers offer further choices on the design and expansion of the material application, like those utilized within the sensors and inductor capacitors [33,34,35,36,37,38].

The weather-shed of out of doors insulators and cable terminators is typically employed by polymeric insulation materials like epoxy and polyethene. Additionally, to their other uses in modern technology, epoxy resins are commonly used as suitable matrices, as they supply flexibility, low shrinking, additives, high dielectric properties, and excellent adhesion. Epoxy blends are quite conventional high-voltage isolators, like epoxy mica composites used to insulate power transformers, station, and line posts, rotary machine isolating coils, and much of more [39,40,41].

In applications, they are not only as inductive and capacitive materials but also microwave absorbers. The distinguishing features of the ferrite/polymer composites are quite desirable. Both features are correlated with sharply lower dielectric losses as compared with bulk ferrites, while because of the advantage of organic, ferromagnetic resonance, the microwave absorption properties remain uninfluenced. Nevertheless, epoxy resins provide excellent electrical insulators and protect the electrical components from short-circuiting, dust, and humidity. Epoxy resins are the primary resin within the industry utilized in integrated circuits, transistors, and hybrids [42].

Composite systems of ferrite nanoparticles with conducting polymers can be used as an active coating for electromagnetic shielding (magnetic and high-frequency) [43,44,45]. This work is devoted to investigating some electrical properties for nickel-zinc lithium ferrite nanoparticles and epoxy/ferrite composites for comparison purposes.

## 2. Materials and Methods

### 2.1. Preparation of Nickel-Zinc Lithium Ferrite Nanoparticles and Epoxy/Ferrite Composites

**Figure 1:**
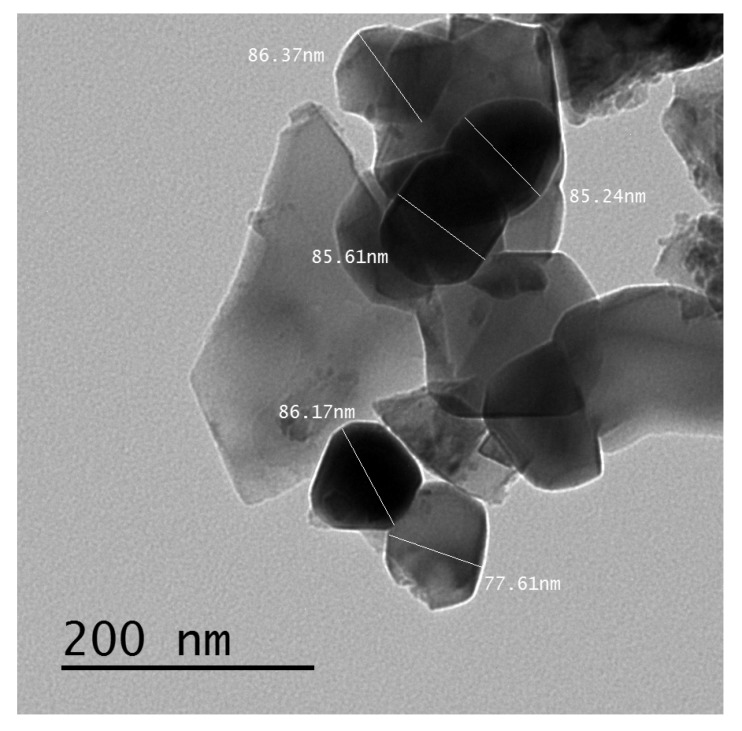
The transmission electron microscopy image for the obtained ferrite nanopowder.

Ferrite nanoparticles with the Li_0.15_(Ni_0.5_Zn_0.5_)_0.7_Fe_2.15_O_4_ (LNCFO) composition were prepared by the chemical co-precipitation method. This specific method for the preparation and characterization of all the samples with the magnetic properties had been previously published [46,47,48,49]. The obtained ferrite powder consisted of particles with an average size in the range from 31 nm to 88 nm. The average crystallite size was determined from transmission electron microscopy data. The particle size has been recalculated by using JEOL JEM-100SX transmission electron microscope (Microscope Unit, Mansoura University, Faculty of Science, Egypt). An illustration of the obtained ferrite nanoparticles and their characteristic sizes are shown in Figure 1.

Then, the ferrite powder was compressed in pellets form for the electrical measurements. The pure epoxy and epoxy/ferrite composites with different ferrite weight contents (20 wt.%, 30 wt.%, 40 wt.%, and 50 wt.%) were cut into pellets for the electrical measurements.

### 2.2. Alternating Current (AC) -Measurements

The AC electrical measurements, such as *σ*_ac_‘ (AC-conductivity) in *S*/cm, *ε*‘ (dielectric constant), and tan*δ* (dielectric loss tangent), were studied with frequencies and temperatures.

In-circuit, the sample coated with conducting material from both sides; in this case, the coating is a silver paste, relates to resistance (*R*) in series. It must be mentioned that the voltage across the resistance must be small enough (≤1%) compared with the root mean square voltage, i.e., the applied voltage (*V*_applied_ = *V*_r.m.s_ = 1 V). The voltage across the resistance (*V_R_*) and the phase angle (*φ*) were measured by using a lock-in amplifier. Here the phase angle (*φ*) is the angle between the *V*_applied_ and *I* = *I*_total_, and the total current is equal to the current across the resistance *R*. The sample used for the electrical measurements can be symbolled as a capacitor with capacitance (*C*) and resistance (*r*). From the equivalent circuit (RC) of the sample, the total current (*I*) is divided into two parts I_c_ and I_r_, and the angle between them is *π*/2. The applied voltage (*V*) can be calculated from the following Equation:

*V* = *V_R_* + *V_C_* (1)

So, *V_C_* >> *V_R_*, we can consider that *V* ≈ *V_C_* and *δ* is the angle between *I* and *I_C_* and named the loss angle or the loss factor.

The following equations were used for calculations of *σ_ac_*′, *ε*′, and tan*δ*.

The next relation can be used to calculate the resistance (*r*) of the sample:

(2) $$r=\frac{V_{C}}{I_{r}}=\frac{V}{I\cos\phi }=\frac{VR}{V_{R}\cos\phi }$$

Since,

(3) $$r=\frac{d}{\sigma_{ac}{}^{\prime }A}$$

where *σ_ac_*′ is the AC-conductivity real part, *d* and *A* are the thickness of the sample (cm), and its surface area (cm^2^), respectively.

So,

(4) $$\sigma_{ac}{}^{\prime }=\frac{d}{rA}=\frac{dV_{R}\cos\phi }{AVR}$$

The reactance of the Resistance-Capacitance RC equivalent circuit of sample *X_C_* is given by the relation:

(5) $$X_{C}=\frac{1}{\omega_{}C}=\frac{V}{I_{c}}=\frac{V}{I\sin\phi }=\frac{VR}{V_{R}\sin\phi }$$

where, ω=2πf*; f* is the frequency of the applied voltage.

(6) $$C=\frac{I\sin\phi }{V\omega }=\frac{V_{R}\sin\phi }{VR\omega }$$

where *C* is the capacitance of the sample, and *V* is the voltage of 1 V.

The capacitance *C_o_* (pF) of an air capacitor consists of two parallel plates of area *A* (cm^2^) and separated by a distance *d* (cm) is given by:

(7) $$C_{o}=\frac{A(cm^{2})}{11.3d(cm)}$$

From the previous equations, the dielectric constant *εʹ* of a sample is:

(8) $$\varepsilon ’=\frac{C}{C_{o}}=\frac{11.3dV_{R}\sin\phi }{RA\omega }\times 10^{12}$$

The dielectric loss is calculated from the *ϕ* phase angle by using the relation:

(9) $$\tan\delta =\frac{1}{\tan\phi }$$

## 3. Results and Discussion

### 3.1. Electrical Properties

#### 3.1.1. The Behavior of Ac Conductivity

This section may be divided by subheadings. It should provide a concise and precise description of the experimental results, their interpretation as well as the experimental conclusions that can be drawn. The following relation gives the complex conductivity (AC):

(10) $$\sigma^{*}(\omega )=\sigma_{ac}^{\prime }+i\sigma_{ac}^{\prime \prime }$$

where the real part of this equation consists of two parts:

(11) $$\sigma_{ac}^{\prime }=\sigma_{1}(T)+\sigma_{2}(T,\omega )$$

The first term, *σ*_1_(*T*) = *σ_dC_*(*T*), is the electrical conductivity produced according to the direct current (DC), and this term is frequency independent and temperature-dependent. *σ*_1_(*T*) follows an Arrhenius, which relates the electric charge carriers and their drift mobility. The second term *σ*_2_(*T*, *ω*) depends on the temperature and the frequency and follows the power law, as shown in the following Equation:

(12) $$\sigma_{2}(T,\omega )=A\omega^{s}$$

where *s* and *A* are parameters that depend on the temperature and the composition [50,51,52]. The power-law relation represents straight lines with slopes equal to the exponent (*s*) and intercepts parts equal to log *A* on the vertical axis at log *ω* = 0. The values of (*T*, at low frequencies are small and increase as *ω* increases, which can have the same explanation that will be discussed regarding the increase of the ac conductivity (*σ*’_ac_) with the frequency and temperature. In the literature, it was also reported that *s* = 0 for *DC* conductivity (frequency-independent) and 0 < *s* < 1 for AC conductivity (frequency-dependent) [53].

**Figure 2:**
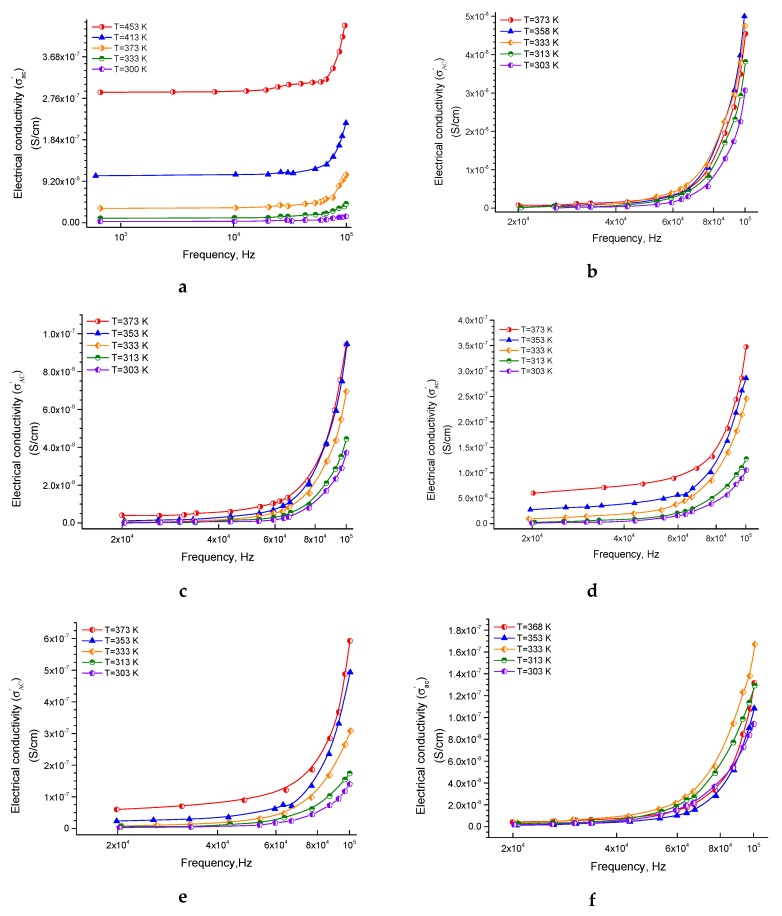
The *σ_ac_*′ electrical AC-conductivity as a function of the frequency of ferrite sintered at 1473K (**a**), pure epoxy (**b**), 20% ferrite + 80% epoxy (**c**), 30% ferrite + 70% epoxy (**d**), 40% ferrite + 60% epoxy (**e**), 50% ferrite + 50% epoxy (**f**).

Figure 2a–f displays the dependence of AC-conductivity (*σ*_ac_′) on frequency in log scale at different temperatures from a range of 300–453K for ferrite, epoxy, and their composites samples. This figure shows that the parameter *σ_ac_*′ of the ferrite sample is usually higher than *σ_dc_*, that was published earlier [51]. This is predictable because *σ_dc_* is a portion of rendering to Equation (11). The *σ_ac_*′ increases with increasing temperature, i.e., increases with increasing frequency at a specified temperature, and this behavior is typical in many kinds of ferrites that have a semiconducting behavior. This type of behavior has been explained according to the hopping conduction mechanism. After increasing the temperature, the electron hopping between Fe^2+^ and Fe^3+^ ions and holes hopping between Ni^2+^ and Ni^3+^ ions increased and led to an increase in the *σ_ac_*′.

Many types of research [54,55,56] have assumed that the ferrites consist of different regions with dissimilar conductivities and have explained the increase in the conductivity of the ferrites with increasing frequency according to Koop’s two-layer model and the space charge polarization [57], recognized to be appropriate to define the heterogeneous structures. It is assumed that the ferrite consists of grains, i.e., functional conductive layers, and grain boundaries, i.e., weak conductive layers. So, as it has been declared above, the conduction takes place by the hopping of electrons and holes. Through hopping, the electron reaching the grain boundary and accumulated there. This occurs because of the high resistivity of the grain boundary and producing so-called space charge polarization [55] that leads to the increase in the dielectric values, as will be discussed later. However, at higher frequencies, the grains were found to be more effective than the grain boundaries and vice versa at lower frequencies [50,51,58].

Figure 2b–f displays *σ*_ac_′ values versus frequency at different temperatures 303, 313, 333, 358, and 373K for the pure epoxy and the composite samples. By comparing the results of *σ*_ac_′ of the pure epoxy and the pure ferrite at almost similar temperatures and frequencies, it has been found that *σ*_ac_′ of the pure epoxy is significantly lower than that of the pure ferrite sample. The pure epoxy has a very low conductivity (very high resistivity), which can decrease the eddy current losses, and this behavior can be appropriate for high-frequency applications [59,60]. This decrease in AC-conductivity is expected, and it is attributed to the more insulating character of the epoxy than that of the ferrite. Whereas in the composites, which are supposed to be composed of islands of ferrites embedded into an insulating matrix of epoxy, it is be expected that of the composite samples will be generally higher than the pure epoxy sample, which has occurred. Also, it may be expected that as the ferrite fraction will increase in the composite, the value of will be increased, which has happened too, except for the composite sample of 50% ferrite. This difference can be clarified by the fact that increasing the number of fillers may increase viscosity, and consequently may cause cracks and defects in the prepared samples. These cracks and defects obviously lower the conductivity. Therefore, a practical recommendation may be concluded here; that is, the suitable fraction of ferrites for preparing such composites is preferred for the low percentage of fillers to avoid cracks and defects. Also, this observation recorded for the pure epoxy in which the non-monotonic behavior concerning the sintering temperature in the pure epoxy may be, in common, due to the dependence of conductivity on several further factors such as the porosity and density [61,62].

Moreover, at lower temperatures, the conduction mechanism may be due to the electron hopping and at higher temperatures may be due to polaron hopping [63,64,65], which means that at high temperature the electron moves by thermal activation and caused a large degree of local lattice distortion so, the mentioned factors can affect the measured results and produce non-monotonicity in the AC conductivity behavior and the other electrical factors.

#### 3.1.2. The Behavior of Dielectric Constant

**Figure 3:**
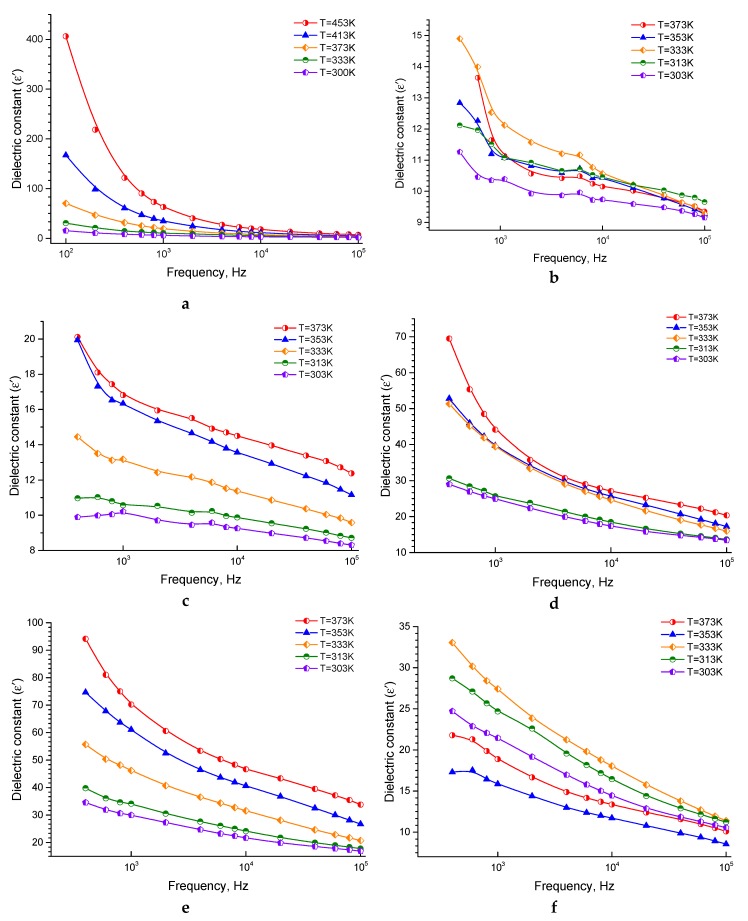
The ε′ dielectric constant as a function of the frequency of ferrite sintered at 1473K (**a**), pure epoxy (**b**), 20% ferrite + 80% epoxy (**c**), 30% ferrite + 70% epoxy (**d**), 40% ferrite + 60% epoxy (**e**), 50% ferrite + 50% epoxy (**f**).

The relation between the dielectric constant (ε′) and the frequency at different temperatures is shown in Figure 3a–f. The ε′ at different temperatures is decreasing with increasing the frequency.

At lower frequencies, the decrease is very sharp, and vice versa, at higher frequencies. The high value of *ε*′ has been explained by Koop’s theory or model [57], as mentioned above. The interfacial polarization occurred according to the accumulation of charges between the grain and grain boundary and increasing ε′ [54,55,56].

Consequently, there is a strong connection between the conduction mechanism, i.e., the conduction process of the ferrites and the dielectric behavior, i.e., the polarization process, which are similar to each other [66]. When increasing the applied field frequency, ε′ decreases and reaches a constant value, because the polarization could not follow its fluctuations at a specific frequency of the applied electric field [67]. Likewise, the electrons direction motion will be reversed by increasing the frequency, and, thus, will prevent the electrons from reaching the grain boundary so that the total polarization will decrease and, as a result, the dielectric constant will also decrease [56].

With increasing the temperature, *ε*′ increased, and this increase can be correctly seen at lower frequencies. With increasing the temperature, the electron exchange interactions increase and, as a result, *ε*′ enhances. It is worth mentioning that the polarization has four contributions in any material—interfacial, dipolar, atomic, and electronic polarization. At high frequencies, atomic and electronic polarizations are necessary and temperature-independent. The interfacial and dipolar polarization are temperature dependent. The interfacial polarization is directly proportional to the temperature, and the dipolar polarization is inversely proportional to the temperature [55,56], and this explains the detected increase in *ε*′ at lower frequencies. From Figure 3b–f, the *ε*′ values of the pure epoxy and composites at almost similar temperatures are significantly lower than that of the pure ferrite sample. This can be explained based on the strong correlation between the conduction mechanism and the dielectric behavior mentioned above, along with the fact that the main contributor to the dielectric constant in such heterogeneous structures is the interfacial polarization. Moreover, the $\varepsilon^{\prime }$ values of the composite samples at almost similar temperatures increase as the fraction of ferrite increases in the sample, except for the sample of 50% ferrite, which may have some cracks as mentioned above.

From the previous discussions, the conductivity results and the constant dielectric results of these samples and their interpretations are consistent and reinforce each other.

#### 3.1.3. The Behavior of Dielectric Loss tangent

**Figure 4:**
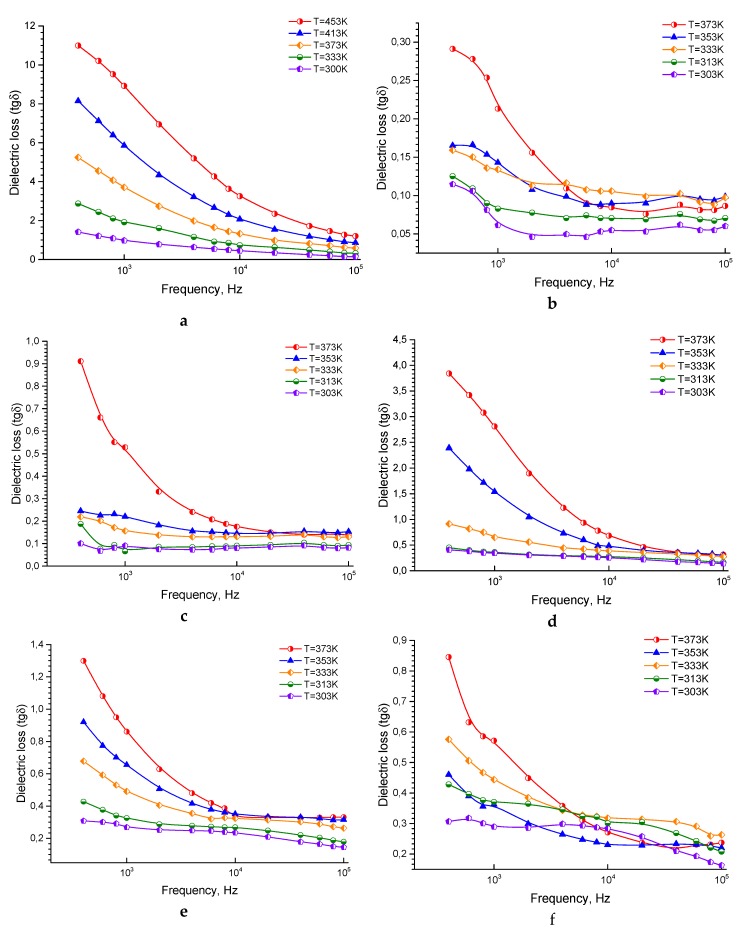
The tan*δ* dielectric loss as a function of the frequency of ferrite sintered at 1473K (**a**), pure epoxy (**b**), 20% ferrite + 80% epoxy (**c**), 30% ferrite + 70% epoxy (**d**), 40% ferrite + 60% epoxy (**e**), 50% ferrite + 50% epoxy (**f**).

The relation between the tan*δ* dielectric loss and the frequency at different temperatures for the ferrite sample, pure epoxy, and the composites is shown in Figure 4a–f. tan*δ* for all the samples shows a typical behavior with frequency, that decreases with increasing the frequency. For all the samples, tan*δ* decreases quickly at low frequencies, although the degree of the decrease is decelerating at the high-frequencies and then turn out to be nearly independent of the frequency. Such behavior can be clarified according to the elevated resistivity due to the grain boundary at the lower frequencies region. So, more energy is essential for the electron exchange between Fe^2+^ and Fe^3+^ ions, and, as a result, the loss is high. Besides, there is a discrepancy with the measured tan*δ* = *ε*″/*ε*′ dielectric loss; where *ε*″, *ε*′ represents the resistive current and the capacitive current, respectively. Due to the grains at the high frequencies (low resistivity), low energy is required for the electron transmission at the octahedral site. Moreover, tan*δ* depends on many factors, such as structural homogeneity, stoichiometry, and Fe^2+^ content. The latter depends on other factors like the annealing temperature and the composition of the samples [54]. Also, as mentioned above, there is a great connection between the conduction mechanism and the dielectric behavior of the ferrites. In *n*-type ferrites, the conduction mechanism is caused by the electrons hopping between Fe^2+^ and Fe^3+^. The hopping occurred at a specific frequency when this frequency is equal to the applied electric field frequency. The dielectric loss is observed to be maximum and very high [66,67,68].

In these samples, the exceptionally high dielectric loss is not observed which is, probably, caused by the low conductivity rendering to the ferrite sample porous structure, and the presence of epoxy in the composite samples may lead to reducing the relaxation frequency of the samples to values less than the measured frequencies range occurred. Also, for all the samples, tan*δ* increases with increasing temperature, which is predictable because the resistivity of the samples decreases with increasing temperature [54]. It is worth mention that the low of loss tangent is observed in pure epoxy may be due to the significant decrease in conductivity according to insulating property of the epoxy so that a decrease of the relaxation frequency of the sample to values less than the measured range of frequencies may have occurred, which may decrease in the loss tangent [69,70,71]. The reduction in conductivity and losses can be useful for several applications demanding the reduction of the eddy current effects [59,60]. The prepared samples are promising materials for practical use as elements of microwave devices according to previous studies depending on the magnetic properties for the studied samples [47,72].

### 3.2. Modeling Dielectric Constant Behavior by Mixing Formulas

To determine the field of application of composite materials (CM), one needs to know their dielectric and magnetic frequency dependence. To solve this problem, it is necessary to describe the dielectric constant of CM as a function depending on the permeability of the primary components: the matrix and the filler.

The standard approach to solving this problem is to apply empirical mixing formulas that use simple idealized models based on a quasi-static consideration of ellipsoidal inclusions and leading to mathematically simple expressions [73,74,75]. As a rule, the two-phases CM is considered, in which identical inclusions are enclosed in a homogeneous matrix (matrix mixtures). The effective properties of such a mixture depend not only on the intrinsic properties of the inclusions and the matrix but also on the morphology of the CM, which characterizes the structure of the material, including concentration, shape, and correlations in the location of inclusions, for example, the presence of agglomerates, the preferential orientation of inclusions, etc. The concentration and characteristics of the form of inclusions usually formulate explicit formulas, and other morphological characteristics are taken into account by choosing its type [76]. There is a considerable variety of mixing formulas. To simulate the dielectric constant, we will use the following ones.

#### 3.2.1. The Maxwell Garnett (MG) Model

This model describes a medium where particles are uniformly distributed in the sample matrix, occupying its entire volume. It is assumed that the interaction between adjacent particles is not significant. We can operate with the concept of the dielectric constant *ε*′ as the total polarizability of all inclusions. The interaction between particles described by this formula is the smallest with the current concentration of inclusions.

(13) $$\varepsilon^{\prime }=\varepsilon_{m}\left(\frac{p}{N\left(1-p\right)+\frac{s_{m}}{s_{i}}}+1\right)$$

where *ε_m_*, *ε_i_* dielectric constant of the matrix and inclusions, respectively; *N* is the form factor of inclusions; *p* – is the bulk density. The transition from mass percentage and density of components to volume are described in [45].

#### 3.2.2. The Symmetric Brüggemann Model

In the literature on magnetic CM [77], it is often called the Polder – van Santen theory. It is based on the formal separation of the CM matrix into individual particles having the same shape as the inclusions; both inclusions and matrix particles are considered immersed in an effective medium characterized by the same permeability as the whole CM. To obtain a homogeneous medium, the polarizabilities of both types of particles must compensate each other, which leads to the equation written concerning the dielectric constant:

(14) $$p\frac{\varepsilon_{i}-\varepsilon^{\prime }}{\varepsilon^{\prime }+N\left(\varepsilon_{i}-\varepsilon^{\prime }\right)}+\left(1-p\right)\frac{\varepsilon_{m}-\varepsilon^{\prime }}{\varepsilon^{\prime }+N\left(\varepsilon_{m}-\varepsilon^{\prime }\right)}=0$$

As a result of solving this equation, we get two roots. The root leading that gives negative ε′ values in plotting was not used.

#### 3.2.3. Weighted Average Model

The value of the dielectric constant of the obtained composite can be found using a simple weighted average [78] of the initial components of the matrix and the material of the inclusions [75]:

(15) $$\varepsilon^{\prime }=\varepsilon_{i}p+\varepsilon_{m}\left(1-p\right)$$

Alternatively, by using the harmonic average formula (model):

(16) $$\varepsilon^{\prime }=\frac{1}{\frac{p}{s_{i}}-\frac{1-p}{s_{m}}}$$

Most of the mixing formulas for the data used to give similar results, so below, we will only consider those that give the greatest agreement with the experiment.

**Figure 5:**
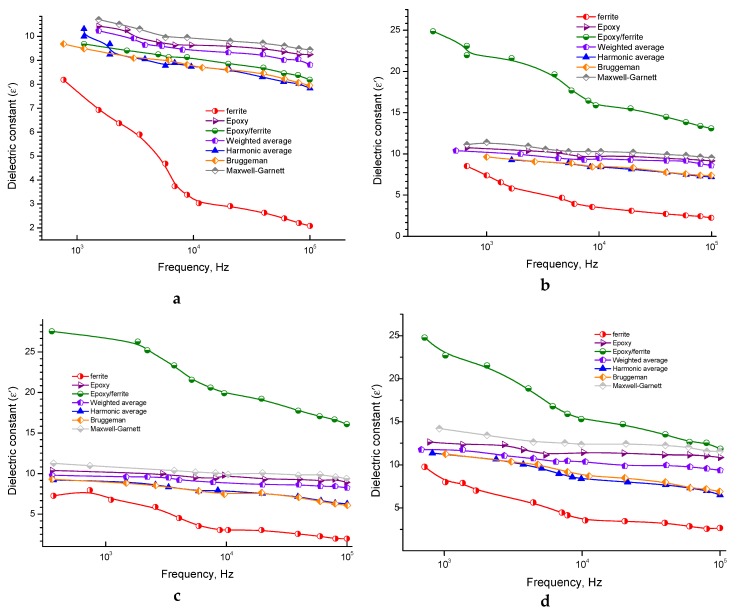
Modeling curves calculated in different approximations for the 20% ferrite + 80% epoxy sample (**a**), 30% ferrite + 70% epoxy (**b**), 40% ferrite + 60% epoxy (**c**), 50% ferrite + 50% epoxy (**d**) at room temperature.

In Figure 5a–d, it can be seen that the values of the dielectric constant for the composite, starting from about 30 wt.%, have a synergistic effect. This is manifested in the fact that the values of *ε*′ for the resulting composite are higher than for the original components.

The research paper [68] describes that the effect of the synergistic increase in permittivity was not observed in composite with high-resistance NiZn fillers, on the other hand, this was observed in composites containing MnZn ferrite that has significant electrical conductivity at high > 40 vol.% filler concentrations. In the considered composites with an even more conductive Li ferrite based on inorganic ferroelectrics, the effect already exists at a low volumetric concentration of fillers. Moreover, this effect was observed in a composite that has manifested fracture with 50 wt.%, although in a more weakened form [79,80].

NiCuZn ferrite, with the addition of TiO_2_, was considered by Yanag et al. 2019 [73]. This additive led to a decrease in the grain size and, accordingly, a more uniform and dense structure of the ferrite. With a smaller grain size, a large part of titanium oxide falls into the intergranular boundaries, forming a new phase there, which increases the resistance of the sample and reduces the losses. Thus, we have a phase of grains in an intergrain medium with high electrical resistance. Such a phase can contribute to the dielectric constant, but this was not taken into account when the model was calculated. In this case, the right way is to apply mixing formulas to filler particles, considering them as a composite in values into the calculated formulas as the contribution of fillers. Therefore, a more thorough study of the structure and elemental distribution in the filler granules may explain the synergistic effect.

The use of the additive ∆ε allows us to obtain the form of a simulated curve with a better approximation order to obtain valid values of the parameters for granules, and already substitute these of the experimental data.

## 4. Conclusions

The AC conductivity for all the samples indicated the presence of the semiconducting behavior in the investigated samples. The AC conductivity of the pure epoxy has a very low value, that can decrease the eddy current losses, and this behavior can be appropriate for high-frequency applications. The AC conductivity of the composites is slightly higher than that of the pure epoxy resin, but still acceptable for practical applications. The permittivity of the composite under consideration was simulated by using various mixing formulas (models). The Harmonic and Bruggeman mixing formulas gave the most significant agreement for the composite with 20 wt.%. Modeling for higher concentrations is challenging to estimate theoretically. This discrepancy could be explained by an increase in the ferrite filler increasing the viscosity, that may cause cracks and non-uniform distribution in the prepared samples. These defects can obviously affect all the measured parameters experimentally, that leads to theoretical results, did not match with the experimental results.
